# Acquired Factor XI Deficiency with Lupus Anticoagulant in a Pregnant Woman Diagnosed by the Eruptions and Pain in Fingers

**DOI:** 10.1155/2020/8854676

**Published:** 2020-12-28

**Authors:** Rie Nakajima, Atsuko Togo, Yasuhira Kanno, Masaru Hayashi, Kanako Mitsuzuka, Hitoshi Ishimoto

**Affiliations:** Department of Obstetrics and Gynecology, Specialized Clinical Science, Tokai University School of Medicine, Japan

## Abstract

We report a case of acquired factor XI deficiency with lupus anticoagulant (LA) in a 28-year-old primigravida who presented with finger pain and eruptions on her palms and fingers during the 3rd trimester of pregnancy. The patient complained of pain and reddening of the fingers at 30 weeks of gestation. She was referred to our tertiary center with a diagnosis of preeclampsia and suspected collagen disease at 35 weeks of gestation. Erythema was seen on the fingers and palms, and she presented with pain and cryesthesia on the fingers. Laboratory investigations revealed an activated partial thromboplastin time of 51 s (normal, 23–40 s), although it was normal during the 30th and 34th gestational weeks, LA with an anticardiolipin-beta2-glycoprotein I complex antibody, and low level of clotting XI activity (25 U/mL). On week 37 day 0 of gestation, the patient presented with severe hypertension. An urgent Cesarean section was performed after transfusion of two units of fresh frozen plasma. There was no excessive bleeding during the surgery or the postpartum period. The symptoms on her fingers and palms gradually improved after surgery. Our case indicates that dermatoses of pregnancy may become a starting point for the diagnosis of autoimmune diseases and coagulation abnormalities. When a patient presents with an atypical symptom, as in our case, the possibility of various diseases should be considered.

## 1. Introduction

Dermatoses of pregnancy often occur during pregnancy and the postpartum period and are sometimes related to autoimmune diseases [1, 2]. Some autoimmune diseases often occur during the reproductive age and are sometimes related to miscarriage or perinatal morbidity [3, 4]. During pregnancy, the coagulation system undergoes significant changes, and coagulation abnormalities are sometimes diagnosed [5, 6]. Factor XI (FXI) is a plasma glycoprotein that acts during the contact phase of blood coagulation. Acquired FXI deficiency is related to autoimmune diseases, malignant tumors, and pregnancy [7–11]. FXI deficiency does not usually lead to spontaneous bleeding but can cause significant bleeding during and after surgery, trauma, and delivery. The risk of bleeding is not clearly related to the FXI levels, which can vary in an individual. Therefore, it is difficult to estimate the risk of bleeding and plan treatment during the intrapartum and postpartum periods. Here, we report a case that was accidentally diagnosed with acquired FXI deficiency in a patient who presented with finger pain and eruptions on palms of the hands and fingers during the third trimester.

## 2. Case Report

**Figure 1:**
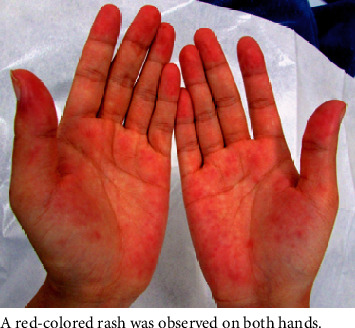
A red-colored rash was observed on both hands.

A 28-year-old primigravida from Cambodia presented at our center. She had no significant past medical or family history. She attended a nearby obstetrics clinic since early pregnancy and developed pain and reddening of the fingers at 30 weeks of gestation. These symptoms gradually aggravated, and her blood pressure increased up to 150/98 mmHg. She was referred to our tertiary center with a diagnosis of preeclampsia and suspected connective tissue disease at 35 weeks of gestation. Her height and weight were 168 cm and 63 kg, respectively. Blood pressure was 140/91 mmHg. Erythema was seen on both the fingers and palms (Figure 1). She complained of pain and cryesthesia on the fingers. The fetus showed normal growth on the ultrasonographic examination. Laboratory data were as follows: white blood cell count, 8.7 × 10^3^/*μ*L; red blood cell count, 4.23 × 10^6^/*μ*L; hemoglobin, 12.9 g/dL; hematocrit, 36.2%; platelets, 16.3 × 10^4^/*μ*L; uric acid, 8.4 mg/dL; and serum creatinine, 0.74 mg/dL. The activated partial thromboplastin time (APTT) was 51 s (normal, 23–40 s), the prothrombin time (PT) was 10.1 s (normal, 9–15 s) [12], and the D-dimer was 2.0 *μ*g/mL (normal in the 3rd trimester, 0.33–3.9 *μ*g/mL) [13] (Table 1). Urinalysis revealed 4+ proteinuria. The APTT was not prolonged during the 30th and 34th gestational weeks. The cross-mixing test was performed on a series of mixtures of patient plasma and normal plasma. APTT tests were performed using patient plasma and normal plasma at five concentration ratios, i.e., 10 : 0, 8 : 2, 5 : 5, 2 : 8, and 0 : 10, in immediate reaction with no incubation, and at three concentration ratios, i.e., 10 : 0, 5 : 5, and 0 : 10, in delayed reaction with 2 h incubation at 37°C [14]. The APTT was not corrected upon mixing with normal plasma in either reaction. The plotting of the results when altering the proportion of normal control plasma to the patient's plasma yielded a convex APTT value curve that faced upward, indicating the presence of circulating anticoagulants including coagulation factor inhibitors and lupus anticoagulant (LA). LA was assessed using the dilute Russell viper venom test. Immunological screening was positive for the anticardiolipin-beta2-glycoprotein I complex antibody (CL-*β*2/GPI). Multiple clotting factor assays revealed a low level of clotting XI activity (25 U/mL) with normal levels of factors II, VIII, IX, XII, and von Willebrand (Table 1) [15]. The patient was diagnosed with factor XI deficiency and suspected antiphospholipid syndrome (APS). At 36 weeks of gestation, the erythema on the fingers became cyanotic. The patient complained of pain and coldness indicative of arterial obstruction on the fingers. Despite the administration of an antihypertensive agent, the blood pressure increased, and protein urea became 4.68 g/day, APTT 51 s, and XI activity 24 U/mL at 36 weeks and 5 days. Simultaneously, fetal growth arrest was suspected on ultrasonography. Urgent delivery was considered. We planned to perform the Bethesda inhibitor assay for the XI factor, but it was not possible because of sudden deterioration of the maternal condition. Given the risk of intrapartum and postpartum hemorrhage, the patient received two units of fresh frozen plasma (FFP) at 36 weeks and 6 days of gestation. On the next day (week 37, day 0 of gestation), the APTT was 50 s. Her blood pressure increased to 180/108 mmHg and could not be controlled with antihypertensive agents. An urgent Cesarean section was performed after blood transfusion. The child was female weighing 2110 g. The Apgar scores were 8 and 9 at 1 and 5 min, respectively. The pH of the arterial cord blood was 7.305. Total blood loss was 683 mL. We planned to transfuse blood on the 3rd postoperative day. However, there was no excessive bleeding after surgery, and we administered FFP on the 1st postoperative day. High blood pressure and finger symptoms gradually improved after the surgery. However, the APTT continued to be prolonged (50 s) and XI activity was 25 U/mL. The placental pathology demonstrated no infarction, perivillous fibrin deposition, and coagulation. The patient was discharged on the 6th postoperative day. At 1 month after giving birth, prolonged APTT (71 s) and low level FXI activity (31 U/mL) were still present. The patient will undergo reexamination for APS 12 weeks later.

## 3. Discussion

We presented the case of a primigravida with acquired FXI deficiency who presented with finger pain and eruptions on the palms and fingers during the 3rd trimester of pregnancy whose symptoms served as a starting point for the diagnosis of low level of coagulation FXI with LA and CL-*β*2/GPI.

Dermatoses of pregnancy often occur during pregnancy and the postpartum period. These include polymorphic eruptions of pregnancy, atopic eruptions of pregnancy, pemphigoid gestationis, and intrahepatic cholestasis of pregnancy [1]. Palmar erythema often occurs during pregnancy with onset in the 1st trimester. This skin condition may be due to vascular changes related to the increased levels of estrogen during pregnancy and is usually not painful. Dermatoses are sometimes related to autoimmune diseases. Autoimmune diseases constitute malfunctions of the body's immune system that cause the body to attack its own tissues. Some autoimmune diseases often occur during pregnancy. Previous studies have reported that hormonal changes, change in T-helper 1/T-helper 2 balance, and microchimerism might be related to the onset of these diseases [3, 4, 7, 8]. APS was suspected in our case; it is characterized by the onset of arterial and/or venous thrombosis. Pregnancy sometimes acts as a trigger for clinical manifestations of APS. Cutaneous manifestations of APS are common, occurring in up to 49% of patients with APS and APS associated with systemic lupus erythematosus (SLE) [9]. They comprise livedo reticularis, ulceration, vasculitis, thrombophlebitis, and subungual splinter hemorrhage [10, 11]. The most common cutaneous manifestation is livedo reticularis, which is significantly associated with ischemic arterial events. A previous report presented the case of a patient with APS who had a painful purpuric rash on the palmar surface during the postpartum period [10]. Biopsy revealed intravascular thrombi. In our case, the patient had similar symptoms suggesting ischemic arterial events. Unfortunately, we could not perform a biopsy. We considered that pregnancy and antiphospholipid antibodies might have been related to the cutaneous manifestations in our case. The risk of thrombosis is higher in the postnatal period than in the antenatal period. However, in our case, the pain and erythema of the patient's fingers and palms gradually improved.

The coagulation system undergoes significant changes during pregnancy. Coagulation abnormalities are sometimes diagnosed during pregnancy [5, 6]. The APTT and PT are used as first-line tests to evaluate the coagulation function. Isolated prolonged APTT can be caused by factor VIII, IX, XI, and XII deficiency or acquired inhibitors of these factors. The cross-mixing test is useful to distinguish between factor deficiencies and the presence of inhibitors. In our case, the cross-mixing test showed an inhibitor pattern that indicated the presence of circulating anticoagulants including coagulation factor inhibitors and LA. Coagulation factor inhibitors are antibodies that neutralize the activity of coagulation factors and increase their clearance, thereby interfering with their normal function as seen in conditions such as hemophilia, autoimmune diseases, malignant tumors, pregnancy, and adverse drug events. LA is an antiphospholipid antibody known to be associated with the risk of thrombosis, as seen in antiphospholipid syndrome. LA inhibits phospholipid-dependent coagulation reactions in the intrinsic, extrinsic, and common pathways. In rare cases, hemorrhagic complications can occur in patients with LA [16]. LA-hypoprothrombinemia syndrome (LA-HPS) is a combination of acquired factor II deficiency and LA and has been reported in conditions including primary antiphospholipid syndrome, infections, and occasionally drug reactions and SLE. LA-HPS might lead to a predisposition to thrombosis and severe bleeding [17]. However, cases of LA with a suspected false-positive test for coagulation factor inhibitors have been reported [18]. For appropriate treatment, careful interpretation of the results of laboratory assays is required in the presence of antiphospholipid antibodies.

FXI is a plasma glycoprotein that participates in the early phase of the blood coagulation cascade and is essential for normal hemostasis. The normal levels of FXI activity range from 70 to 150% [19, 20]. The change in FXI levels during pregnancy is inconsistent, and studies have reported slight increase [21], no change [22], or decrease [23]. The diagnosis of severe FXI deficiency is defined as FXI activity < 15% of normal, and individuals with partial FXI deficiency usually have FXI activity between 20 and 70% of normal [22]. Those in the higher activity range can have a normal APTT. Acquired FXI deficiency is mainly seen in patients with liver disease due to production failure or following the onset of disseminated intravascular coagulation. In addition, cases of FXI deficiency following autoimmune disease, malignant disease, and pregnancy have been reported [24]. FXI inhibitors are rare and have been reported in patients with severe congenital FXI deficiency following plasma infusion or in autoimmune diseases such as SLE [25]. The development of autoantibodies against coagulation factors occurs in 10 to 15% of patients with SLE. Women with FXI deficiency are prone to bleeding following surgery, trauma, and delivery [26, 27]. The risk of intrapartum and postpartum hemorrhage has been reported by numerous case reports; it is variable and might have no relationship with the FXI level or history of bleeding [19]. Davies and Kadir found that the reported incidence of primary postpartum hemorrhage (PPH) in women with FXI deficiency ranges from 10 to 22% with 85 cases reported in 490 deliveries (mean incidence, 17%). This is significantly higher than the incidence of primary PPH (5–8%) in the general obstetric population [28]. Treatment for intrapartum and postpartum hemorrhage includes replacement therapy with FFP (15 to 20 mL/kg per day for 2 to 4 days) or FXI concentrates, antifibrinolytic agents, and low-dose recombined human factor VIIa (rFVIIa). Low-dose rFVIIa is used in patients with FXI inhibitors. However, low-dose rFVIIa and FXI concentrates are not approved in our country because of their thrombotic complications. There is no consensus on the routine need for replacement therapy in patients with severe FXI deficiency at the time of delivery and during the postpartum period [29–31]. In our case, pregnancy and LA might have been associated with the low level of FXI. The prolonged APTT did not change after administration of FFP; hence, we suspect that an FXI inhibitor was present. The lack of an inhibitor assay for FXI should be noted as a limitation of this case report. We selected the treatment considering the risks of bleeding and thrombosis. Appropriate treatment is required according to the patient's condition during delivery and the postpartum period.

In our case, the erythema on the fingers and palms was a starting point for the diagnosis of low level of coagulation FXI with LA and CL-*β*2/GPI. Many physiological changes are seen during pregnancy. If a patient presents with an atypical symptom, similar to that in our case, the possibility of various diseases should be considered.

In conclusion, dermatoses during pregnancy are sometimes related to autoimmune diseases. Painful erythema on the fingers and palms is a symptom that requires attention during pregnancy.

## Figures and Tables

**Table 1:** Laboratory results of coagulation studies.

Parameters	Patient value	Normal range
Coagulation screening profile		
APTT (seconds)	50	23-40
PT (seconds)	10.1	9-15
INR (*μ*g/mL)	0.85	0.80-0.94
D-dimer (*μ*g/mL)	2.0	0.33-3.90
Coagulation factor assay		
Factor VIII (%)	99	50-200
Factor IX (%)	94	50-150
Factor XI (%)	25	65-123
Factor XII (%)	123	70-145
von Willebrand factor (%)	254	67-336

APTT: activated partial thromboplastin time; PT: prothrombin time; INR: international normalized ratio/the normal ranges are based on the values provided in References [12, 13, 15].
